# Identification of Novel and Efficacious Chemical Compounds that Disturb Influenza A Virus Entry *in vitro*

**DOI:** 10.3389/fcimb.2017.00304

**Published:** 2017-06-30

**Authors:** Hany Khalil, Tamer El Malah, Ahmed I. Abd El Maksoud, Ibrahim El Halfawy, Ahmed A. El Rashedy, Mahmoud El Hefnawy

**Affiliations:** ^1^Department of Molecular Biology, Genetic Engineering and Biotechnology Research Institute, University of Sadat CitySadat, Egypt; ^2^Photochemistry Department, National Research CentreGiza, Egypt; ^3^Industrial Biotechnology Department, Genetic Engineering and Biotechnology Research Institute, University of Sadat CitySadat, Egypt; ^4^Department of Molecular Diagnostics, Genetic Engineering and Biotechnology Research Institute, University of Sadat CitySadat City, Egypt; ^5^Natural and Microbial Department, National Research CenterGiza, Egypt

**Keywords:** influenza A virus, drug discovery, cell culture, organic compounds, virus entry

## Abstract

Influenza A virus is a negative RNA stranded virus of the family *Orthomyxoviridae*, and represents a major public health threat, compounding existing disease conditions. Influenza A virus replicates rapidly within its host and the segmented nature of its genome facilitates re-assortment, whereby whole genes are exchanged between influenza virus subtypes during replication. Antiviral medications are important pharmacological tools in influenza virus prophylaxis and therapy. However, the use of currently available antiviral is impeded by sometimes high levels of resistance in circulating virus strains. Here, we identified novel anti-influenza compounds through screening of chemical compounds synthesized *de novo* on human lung epithelial cells. Computational and experimental screening of extensive and water soluble compounds identified novel influenza virus inhibitors that can reduce influenza virus infection without detectable toxic effects on host cells. Interestingly, the indicated active compounds inhibit viral replication most likely via interaction with cell receptors and disturb influenza virus entry into host cells. Collectively, screening of new synthesis chemical compounds on influenza A virus replication provides a novel and efficacious anti-influenza compounds that can inhibit viral replication via disturbing virus entry and indicates that these compounds are attractive candidates for evaluation as potential anti-influenza drugs.

## Introduction

Influenza A virus (IAV) belongs to the family *Orthomyxoviridae*, and is considered as one of the most dangerous viruses that threaten human entity and causing up to 500,000 deaths per year worldwide. The threat posed is further intensified by its potential to cause pandemics, for instance, the “Spanish Flu” of 1918–1919, which resulted in ca. 40 million deaths, and the recent emergence of the influenza A (H1N1) “swine flu” strain (Claas et al., 1998; Subbarao et al., 1998; Fraser et al., 2009). IAV has a single-stranded, negative sense RNA genome (Neumann et al., 2004), which is distributed over eight RNA strands. The structure of the IAV is somewhat changeable; however, the virus particles usually have 80 to 120 nanometers in circular shape. Sometimes filamentous forms of the virus occur as well, and are more common among some influenza strains than others (Bouvier and Palese, 2008). The influenza virion is an enveloped virus particle that derives its lipid layer from the plasma membrane of a host cell. Two different varieties of glycoprotein prickles are surrounded in the envelope. Approximately 80% of the prickles are hemagglutinin (HA), a trimeric protein that functions in the attachment of the virus to a host cell (Lamb and Choppin, 1983). The remaining 20% or so of the glycoprotein prickles consist of neuraminidase (NA), which is thought to be mainly involved in facilitating the release of virus progeny from the host cell. On the inner side of the envelope that surrounds influenza virion is an antigenic matrix (M) protein coating. Within the envelope is the influenza genome, which is organized into eight segments of negative-stranded RNA (A and B forms only; influenza C has 7 RNA segments) encode to eleven different proteins (Lamb and Choppin, 1983; Bouvier and Palese, 2008). The RNA is packaged with nucleoprotein into a helical ribonucleoproteins form, with three polymerase peptides for each RNA segment (Fujiyoshi et al., 1994; Holmes et al., 2005). IAV replicates rapidly within its host and the segmented nature of its genome facilitates reassortment, whereby whole genes are exchanged between IAV subtypes during replication. This can result in large and rapid antigenic shift, whereby new IAV subtypes develop suddenly and randomly, to which the human population may have little or no immunity. This poses particular challenges for vaccine development; vaccines must be developed rapidly in response to the emergence of strains with pandemic potential. As such, antiviral medications are key therapeutic and prophylactic weapons. Currently available antiviral medications target the viral proteins matrix 2 (M2) and neuraminidase (NA). Amantadine and remantadine target the viral M2 protein and can help to reduce severity of illness in individuals with IAV when treatment is initiated within 2 days of the onset of symptoms. These drugs bind to M2, a proton ion channel, to inhibit the change in pH that is necessary for the influenza virion to release its contents into the cytosol of a host cell (Cyranoski, 2005). Two additional antiviral drugs, zanamavir (Relenza) and oseltamivir (Tamiflu) are effective against subtypes of both IAV and Influenza B virus. These compounds inhibit the viral glycoprotein, neuraminidase, so that the release of new virus particles is inhibited and spreading infections are limited (Ward et al., 2005; Yen et al., 2007). Antibiotics also frequently play a role in influenza treatment interventions but only for the management of opportunistic secondary infections, such as bacterial pneumonia. Whilst pharmacological interventions are the key importance in influenza therapy and prophylaxis, resistance to the majority of currently available antiviral now limits their utility, and in some cases precludes it entirely (amantadine). The IAV H1N1 seasonal 2009 strain acquired a neuraminidase mutation, such that oseltamivir resistance is now widespread in this strain. Therefore, it became necessary to stockpile oseltamivir alongside additional drugs, including zanamavir, during the H1N1 pandemic to increase the efficiency of its antiviral activity against H1N1 and limit the emergence of resistance (Ward et al., 2005). Noteworthy, over-use of antiviral is considered a factor for development of viral drug resistance, in the case of amantadine, drug treatment leads to rapid influenza virus resistance. The high level of resistance to amantadine in circulating influenza viruses may be due to the availability of amantadine as a part of over-the-counter cold medicines (Cyranoski, 2005). Recently, some other effective anti-influenza inhibitors have been identified such as ANA-0 that suppresses viral PA endonuclease activity resulted in protection of mice from lethal dose and decrease of lung viral loads (Yuan et al., 2016). The thiol antioxidant glutathione (GSH) showed an inhibition of IAV replication via targeting matrix protein and subsequently modulation of viral-inducing apoptotic signaling (Cai et al., 2003). By inhibition of viral sialidase, glucosyl hesperidin can also prevent IAV replication (Saha et al., 2009). Interestingly, genome wide RNAi screens have revealed that many host cell factors are essential for the replication of IAV (Hao et al., 2008; Karlas et al., 2010; Konig et al., 2010). These factors are attractive candidates for potential antiviral medications as it is less likely that influenza viruses will develop resistance rapidly to drugs that target host cell factors. The current work aims to identify novel antiviral compounds that are active against IAV, H1N1 strain. Potentially active water soluble compounds were synthesized *de novo*. The antiviral activity of these compounds were assessed using a global screening approach in human lung epithelial cells (A549 cell line) infected with IAV. Screening of these compounds reveals some attractive candidates that successfully reduced viral replication without any detectable toxic effect most likely via disturbing viral entry.

## Methods

### Synthesis of chemical compounds

Chemical synthesis was initiated by selection of compounds that are recognized as reactants and their mixing in a reaction vessel. Different reactions were used to synthesize the transitional or final products. Ultimately, the amount of product correlates with the reaction yield, with chemical yields expressed as weight in grams or as a percentage of the total quantity of product created (Fillon et al., 2003).

### Cells lines and influenza virus

Influenza virus strain A/WSN/33 (H1N1) were propagated in the allantoic membrane of embryonated chicken eggs by injecting 11-day-old eggs with virus and then incubating the infected embryos to allow virus growth. High titers of viruses were then recovered from the infected eggs (Karlas et al., 2010). Human lung epithelial cells (A549 cells) (CCL-185, ATCC-LGC, Wesel, Germany) were grown in DMEM media (Invitrogen, Karlsruhe, Germany) supplemented with 4 mM L-glutamine, 4 mM sodium pyruvate, 100 U/ml penicillin/streptomycin and 10% fetal calf serum (FCS), at 37°C and 5% CO_2_. Human embryonic kidney cells (293T cells) (CRL-11268, ATCC-LGC), and Madin-Darby canine kidney cells (MDCK cells) (CCL-34, ATCC-LGC) were grown in DMEM supplemented with 4 mM L-glutamine, 100 U/ml penicillin/streptomycin and 10% FCS.

### Chemical treatment and virus infection

To screen the efficiency of organic chemical compounds on IAV replication, A549 cells were seeded in 96-well plate. 24 h later, cells were treated 2 h prior infection with different concentrations of each compound or left without treatment. The treated cells were then infected with IAV (MOI equal 0.05) for 1 h at room temperature following by overnight incubation with the same concentrations of each compound.

### Virus quantification

The infectious virus particles in supernatant was quantified by using a virus dependent luciferase assay which previously described by Lutz et al. (2005) and traditional plaque assay. The virus-dependent luciferase reporter construct (Flu-Luci) has been performed by using RNA polymerase I promoter/terminator cassette to express RNA transcripts encoding firefly luciferase flanked by the untranslated regions of the influenza A/WSN/33 nucleoprotein (NP) segment. Based on the ability of IAV to drive luciferase production in Flu-Luci transfected 293T cells, the luciferase assay was carried out to assess viral infection (Lutz et al., 2005). Accordingly, the Flu-Luci reporter construct was used to transfect 293T cells, and then transfected cells were seeded into black 96-well plates at concentrations of 1 × 10^4^/well. Twenty four hours later, cells were infected with 50 μl of the supernatant of virus-infected cell cultures. At 16 h post-infection (h p.i.), cells were lysed with “passive lysis buffer” (Promega, Madison, WI, USA) and luciferase activities in resulting cell lysates were monitored with a firefly luciferase assay using firefly substrate, as previously described (Dyer et al., 2000). For plaque assay, MDCK cells were seeded in 6-well plates at a concentration of 1 million cells per well. Twenty four hours later, cells were infected with six different dilutions of the supernatant of virus-infected cells culture. One hour post-infection, the infectious media was removed and 2 ml of 2x MEM media contains 500 μl 10% agar solutions, 5 μl dextrane and 5 μl BSA was added to each well. The infected cells were incubated for 2 days and then were fixed overnight with PBS contains 3.7% formaldehyde. For staining, the agar layer was removed and cells were stained with 0.1% crystal violet dissolved in 20% ethanol. The virus plaques were counted manually and plaque forming units per ml was calculated.

### Cytotoxic effect and metabolic activity of host cells

Assessing of lactate dehydrogenase (LDH) released into the media of treated cells was monitored in 96-well plate using LDH detection kit. According to the manufacture procedures, 40 μl of samples was incubated with 40 μl LDH buffer and 20 μl LDH substrate for 1 h then the relative LDH production was calculated according to the standard curve. Cells treated with 50 and 100 μl of Triton x-100 served as a positive control. Further, immunoflorescent images of treated cells that were stained with DAPI and accounting of living cells upon treatment were used to investigate cytotoxic effect of each individual compound.

### Western blot

Immunoblotting of IAV-NP protein was performed by using the vertical Mini-Protean II electrophoresis system (Bio-Rad). Equal amounts of total proteins were loaded into 12% polyacrylamide gel electrophoresis. Then the separated proteins were transferred onto PVDF membranes (Bio-Rad) that activated by methanol for 1 min. The membranes were incubated for 1 h at 37°C with blocking buffer (PBS with 5% non-fat-milk). Then, the membranes were incubated overnight at 4°C with mouse monoclonal anti-influenza NP (AbD Serotech, UK) or Mouse monoclonal anti-β-actin (Sigma, Hamburg, Germany) in dilution buffer (1–1,000). Proteins profile were detected with 1–10,000 diluted sheep anti-mouse secondary antibody conjugated with horseradish peroxidase enhanced chemi-luminescence system (ECL, Amersham). β-actin was used as a loading control.

### Fluorescence confocal microscopy

A549 cells were plated onto cover slips in 24-well plates at a density of 2 × 10^4^ cells per well and were incubated overnight. Cells were then treated for 2 h prior infection with 50 μg/ml of the indicated chemical inhibitors or left without treatment. Subsequently, cells were inoculated with IAV at MOI of 0.05 and incubated at 37°C and 5% CO_2_. At 24 h p.i., cells were fixed using 4% paraformaldehyde for 25 min at room temperature (RT), permeabilized with cold methanol for 10 s and incubated overnight at 4°C with a primary monoclonal antibody against influenza NP (1:500 dilution). Cells then were washed three times with PBS were sequentially incubated in the dark with Cy5-conjugated monoclonal anti-NP antibody (clone: AA5H, AbD Serotech, Oxford, UK; 1:100 dilution). Finally, cells were washed again three time using PBS and were stained with the fluorescent DNA dye DAPI at 1 μg/ml for 15 min. After washing the stained cells, the florescent images were captured using a laser scanning confocal fluorescence microscope with a 10X objective (Olympus FluoView FV10i) (Khalil et al., 2016; Tazi et al., 2016).

### Quantitative RT-PCR

Total RNA from treated and infected A549 cells (MOI of 1) was extracted in different time point upon infection (2, 4, and 8 h p.i.) and purified using TRIzol (Invitrogen, USA) and the RNeasy Mini Kit (Qiagen, USA). Quantitative RT-PCR (qRT-PCR) was used to detect the relative expression of IFN-β in A549 cells upon influenza A virus infection by using the QuantiTect SYBR Green PCR Kit (Qiagen) and the following oligonucleotides specific IFNβ1_for:5′-CAG CTC TTT CCA TGA GCT AC-3′ and IFNβ1_rev:5′-CAG CCA GTG CTA GAT GAA TC-3′. Levels of GAPDH were amplified using specific oligonucleotides, GAPDH-For-5′-TGGCATTGTGGAAGGGCTCA-3′ and GAPDH-Rev-5′-TGGATGCAGGGATGATGTTCT-3′ which was used for normalization. The following parameters have been used in RT-PCR program, 95°C for 5 min, 35 cycles (94°C for 45 s, 60°C for 30 s and 72°C for 30 s) and finally 72°C for 10 min. The results were analyzed using ΔΔ Ct equations (Schmittgen et al., 2004; Khalil et al., 2017).

### Elisa

IFN-β secretion after IAV infection was assessed using the human IFN-β Elisa Kit (Thermo Scientific, 414101), following the manufacturer's instructions. Briefly, A549 cells were seeded into 96-well-plates at a concentration of 10 x 10^3^ cells/well. Cells were pre-treated with the indicated concentration of chemical inhibitors or left without treatment and then infected with IAV (MOI 1) for 2, 4 or 8 h. Supernatants were used to measure the concentration of secreted IFN-β using a micro-plate reader (450 nm). Results were processed using Soft-Max program V5 (Molecular Devices, CA, USA).

### Docking analysis

The binding affinity of synthesized compound EMT-305 and EMT-104 and hemagglutinin receptor was analyzed using molecular docking investigation to find out the possible interactions between ligand and receptor. For the docking calculations, the protein structure (PDB code: 1HGI) was first separated from the inhibitor molecule and refined using molecular minimization with added hydrogen (Sauter et al., 1992). Docking calculations were carried out using standard default variables for the MOE program. The binding affinity was evaluated by the binding free energies (S-score, kcal/mol), hydrogen bonds, and RMSD values. Both indicated compounds were docked into same groove of the binding site of the native co-crystallize ligand. The Dock scoring in MOE software was done using London dG scoring function and has been enhanced by using two different refinement methods, the Force-field and Grid-Min pose which have been updated to ensure that refined poses satisfy the specified conformations. We allowed rotatable bonds; the best 10 poses were retained and analyzed for the binding poses best score. Energy minimization was done through Force-field MMFF94x Optimization using gradient of 0.0001 for determining low energy conformations with the most favorable (lowest energy) geometry.

### Statistical analysis

Microsoft Excel was used for statistical calculation, graphs, and histograms. Student's two tailed *t*-test was used to determine significance of different values indicated by luciferase activity and plaque forming units in infected and pre-treated cells. ImageJ software was used in quantitative analysis of viral NP positive cells in microscope images (Khalil et al., 2016). SDS 2.2.2 software was used to analyze the qRT-PCT data to drive the ΔΔCt values using the following equations; (ΔCt) = Ct for gene – Ct for GAPDH. (ΔΔCt) = ΔCt for sample – ΔCt for control. Finally, the relative gene expression is equal 2^−ΔΔct^ of the final values (Schmittgen et al., 2004; Khalil et al., 2016).

## Results

### Detection of chemical compounds with antiviral activity against IAV replication

**Figure 1:**
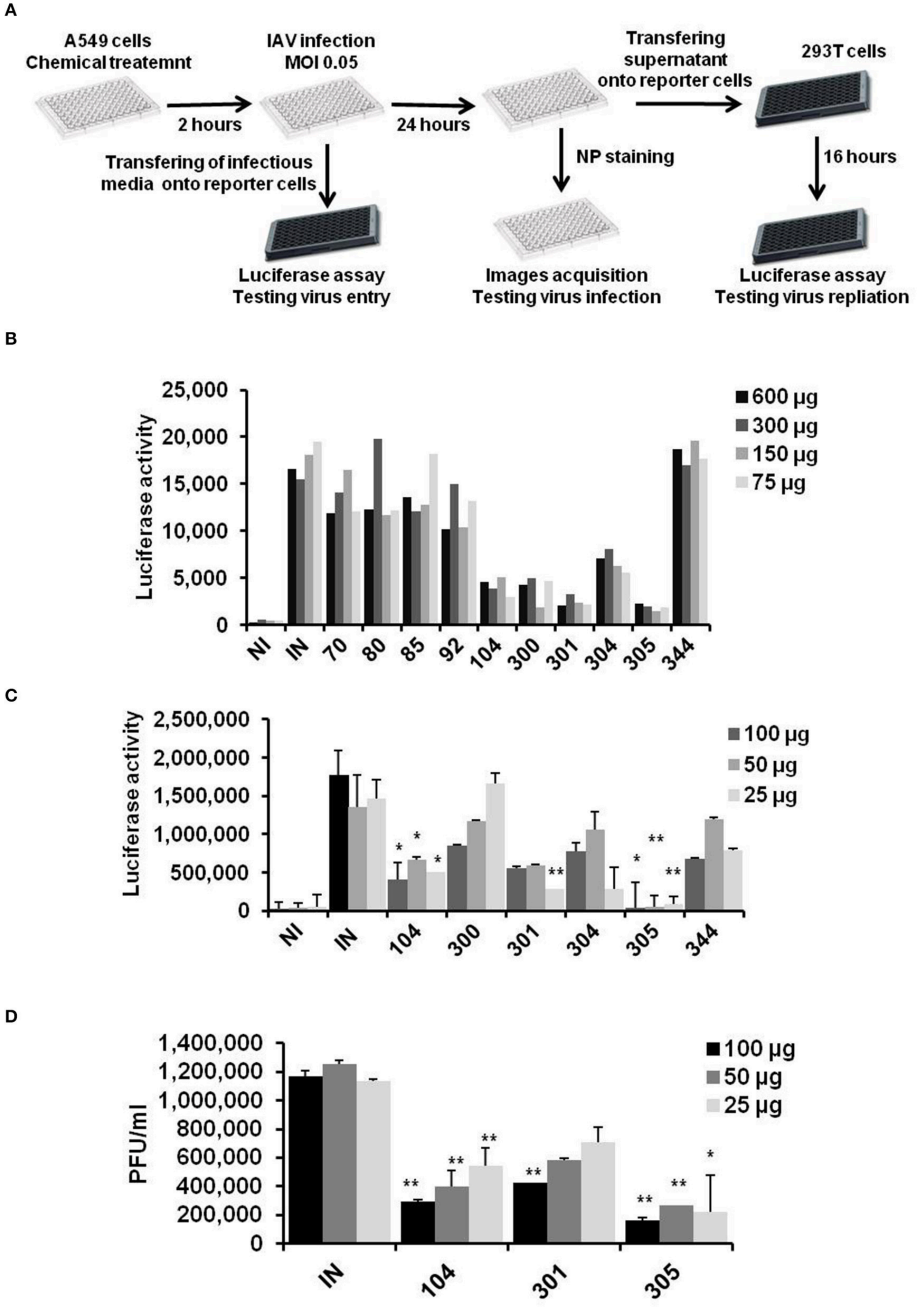
Chemicals screening on influenza A virus replication. **(A)** Schematic representation of chemical inhibitors screening on infected A549 cells using 96 well plates as primary infection. Infection buffer and infectious media were used to infected 293T cells to determine remained virus and produced viral particles, respectively, using virus dependent luciferase assay. **(B)** Global screening of chemical inhibitors that are synthesis *de novo* on IAV replication using virus-dependent luciferase assay. A549 cells were pre-treated with the indicated concentrations of chemical inhibitors and were infected with IAV (MOI of 0.05). 24 h post-infection, infectious media was collected and used to infect 293T cells that were transfected with Flu-Luci plasmid for 24 h. The luciferase activity reveals the virus particles and replication activity of IAV upon infection of 293T cells. **(C)** Relative luciferase activity on 293T cells infected with infectious media that was collected 24 h upon infection of pre-treated A549 with the indicated inhibitors. Error bars indicate the standard deviation (*SD*) of three different replication **(D)** Plaque forming units of virus particles on MDCK cells infected with the infectious media that was collected 24 h upon infection of A549 cells. Students two-tailed test was used to determine the significance values of viral replication. ^*^P < 0.05; ^**^P < 0.01. Error bars indicate the standard deviation of two independent experiments.

Detection of anti-influenza compounds was initiated with a global screening of several water soluble compounds for their ability to reduce IAV, H1N1 strain, replication. In this screening, A549 cells seeded in 96-well plates were pre-treated with indicated concentrations of chemical compounds. The treated cells were infected with IAV (MOI of 0.05), for 1 h at RT. Next, the infection buffer was collected and immediately used to infect 293T cells that were transfected with an established virus-dependent luciferase construct to detect the remaining viruses and investigate the possible interruption of virus entry during primary infection (Lutz et al., 2005). Furthermore, pre-treated and infected A549 cells were used to quantify the virus particles in a secondary infection by using MDCK and transfected 293T cells. Finally, A549 cells were fixed and stained for detection of viral nucleoprotein (NP) protein expression (Figure 1A). The preliminary results indicated by luciferase screening showed strong reduction of virus particles in A549 cells that overnight incubated with compounds EMT-104, EMT-300, EMT-301, and EMT-305 compared to infected cells (IN) and noninfected cells (NI) (Figure 1B). To further confirm the anti-viral activity of these compounds, additional luciferase assay and traditional plaque assay were used to monitor virus particles in cells that overnight incubated with the concentrations of 100, 50, and 25 μg from each compounds. Interestingly, the luciferase activity in treated cells reveals significant inhibition of IAV in cells that treated with different concentrations of EMT-104 and EMT-305 (Figure 1C). Meanwhile, plaque forming units per/ml in treated cells exhibit further competitive inhibition of IAV replication in cells treated with compounds EMT-104 and EMT-305 (Figure 1D). These results indicate that these compounds are attractive candidates for evaluation as potential anti-influenza drugs.

### The preferred compounds have no cytotoxic effects in cell viability rate

**Figure 2:**
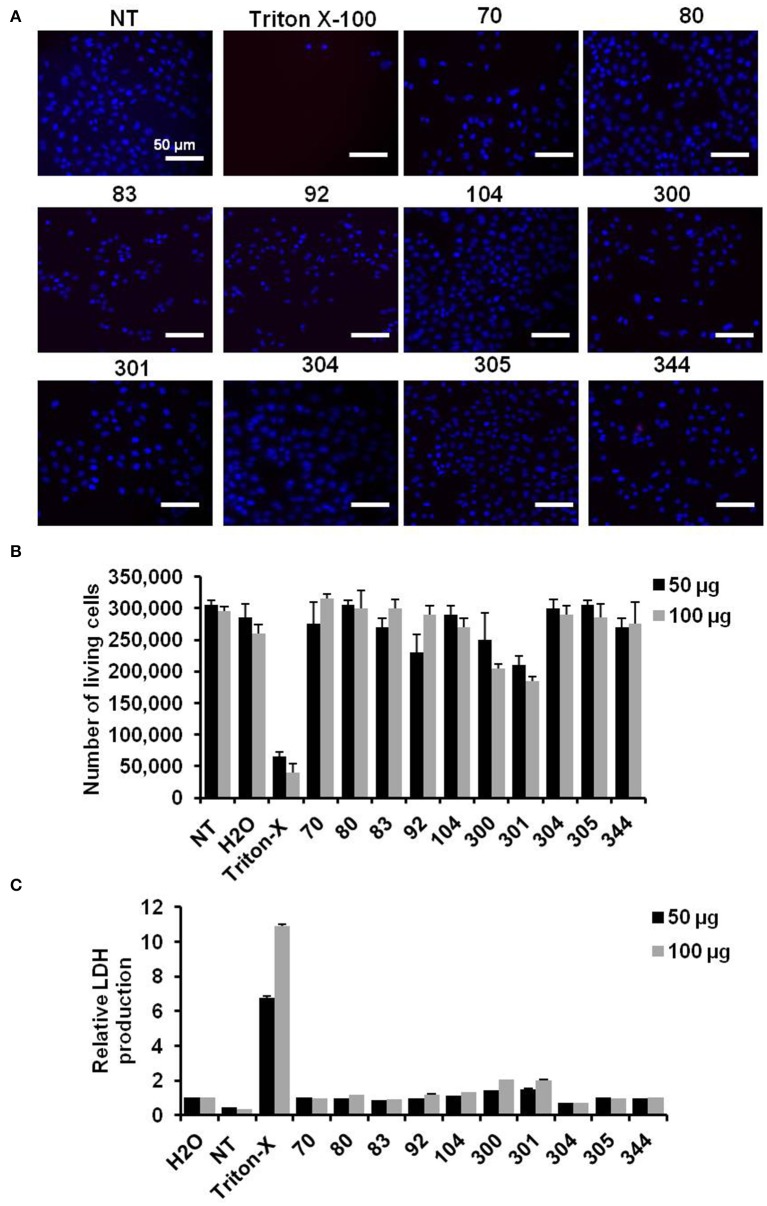
Cell viability and toxic effect of chemical compounds. **(A)** Images reveal cell viability of A549 cells that are pre-treated with the indicated inhibitor and infected with IAV for 24 h in comparison with untreated cells (NT) and cells pre-treated with Triton X-100. **(B)** Number of A549 cells pre-treated with the indicated inhibitors and infected with IAV in comparison with NT cells, Triton X-100, and water treated cells. **(C)** Relative LDH production of pre-treated and infected A549 cells reveals the cytotoxic effect of the indicated inhibitors. Error bars indicate *SD* of three independent experiments.

The influence of chemical compounds on cell viability rate was monitored depending on cell imaging and number of living cells following incubation. Additionally, lactate dehydrogenase (LDH) production from treated cells was measured as an indicator for cytotoxic effect of active compounds. Cells imaging and number of living cells showed no detrimental influence on treated cells with the indicated chemical compounds compared with cells treated with the Triton X-100, as detergent, or cells that left without treatment (NT) (Figures 2A,B). Further, relative LDH production on treated cells showed a negligible cytotoxic effect particularly in cells treated with active compounds against IAV replication (Figure 2C). Taken together, these data indicate that the water soluble compounds EMT-104 and EMT-305 strongly inhibit IAV replication without any detectable cytotoxic effect.

### The indicated inhibitors possess the antiviral activity via disturbing virus entry

**Figure 3:**
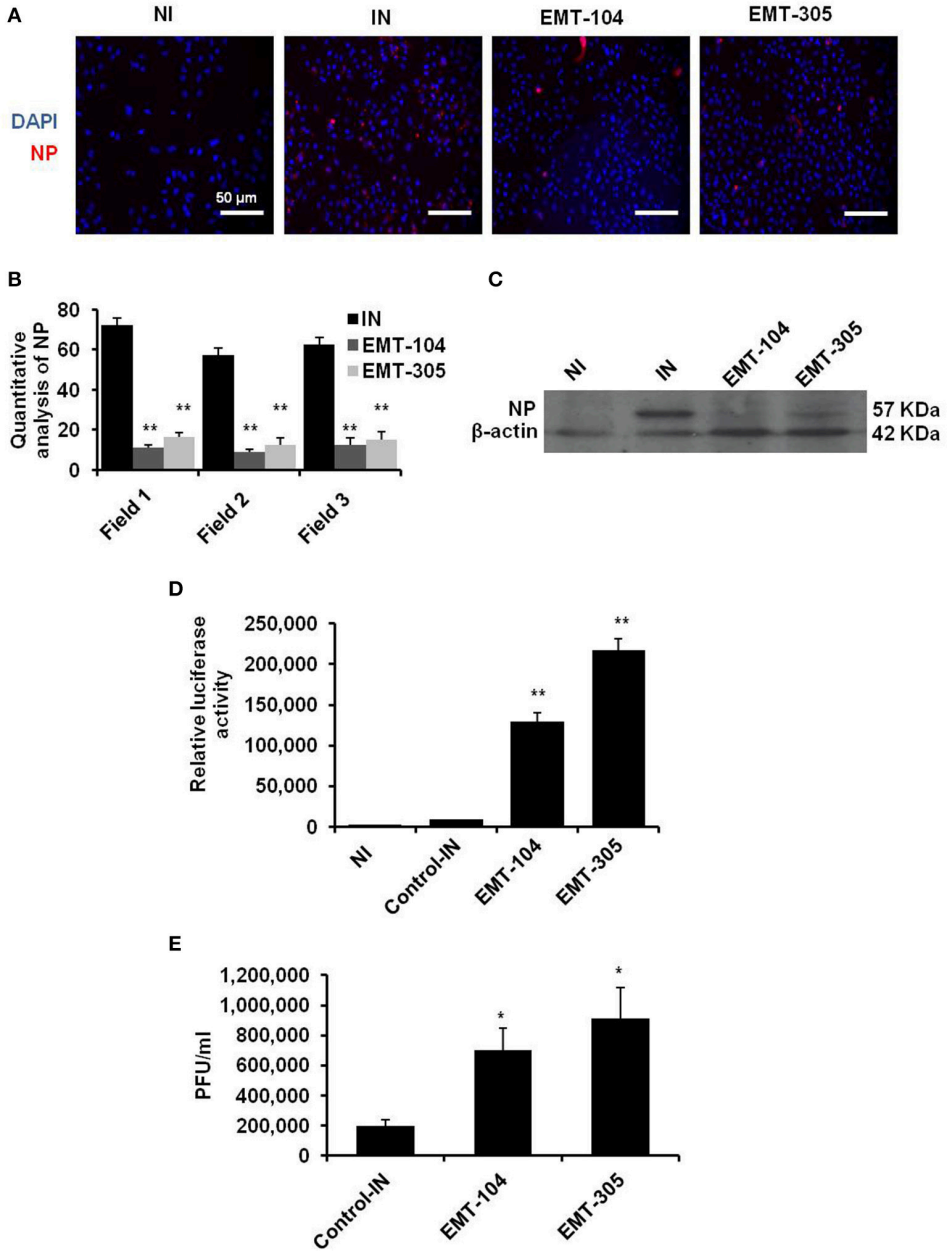
EMT-305 and EMT-104 compounds inhibit IAV infection via disturbing entry. **(A)** Representative confocal images depicting infected A549 cells revealing the expression of viral NP (red) and DNA (blue). **(B)** Quantitative analysis of viral-NP positive cells using ImageJ software. Students two-tailed test was used to determine the significance of NP positive cells. Data is representative of two independent experiments. ^*^P < 0.01. Error bars indicate the *SD*. **(C)** Western blot analysis of viral NP protein in infected and pre-treated A549 cells. β-actin served as loading control. **(D)** Relative luciferase activity of infected 293T cells reveals the remaining viral particles on the infectious media that was collected 1 h post-infection of A594 cells pre-treated with the indicated inhibitors. **(E)** Plaque forming units of viral particles on MDCK cells infected with infectious media that was collected 1 h post-infection of A594 cells pre-treated with the indicated inhibitors. Error bars indicate *SD* of three independent experiments. Students two-tailed test was used to determine the significance of remained virus particles (^*^P < 0.05; ^**^P < 0.01).

Influenza virus NP is a structural protein bind to negative strand RNA in viral nucleocapsid. Together with viral RNA polymerase proteins, NP protein is essential and necessary to catalyze transcription of negative strand RNA to positive uncapped mRNA segments and translation of viral proteins. Other evidences indicate the crucial role of NP protein during viral replication through interaction with cellular factors such as autophagy and retinoic acid-inducible gene 1 (RIG1) proteins (Pichlmair et al., 2006; Khalil, 2012). Thus, the expression level of NP protein reveals the ability of IAV replication in infected cells. To further confirm the effectiveness of selected inhibitors on viral replication, the expression of viral NP protein was monitored in pre-treated cells by using florescent antibody. The protein level of viral NP has been reduced in infected A549 cells that pre-treated with EMT-104 and EMT-305 inhibitors in comparison with infected cells (IN) and noninfected cells (NI) (Figure 3A). The quantitative analysis of florescent NP was quantified using ImageJ 1.48 software. The quantification demonstrates that NP positive cells was significantly reduced in pre-treated cells in comparison with untreated and infected cells (IN) (Figure 3B). Furthermore, the expression of corresponding protein was also reduced as demonstrated by immunoblotting assay with specific antibodies to viral NP protein (Figure 3C). To investigate whether selected compounds have an effect on virus entry, the infection buffer used in primary infection was collected upon 1 h post-infection of pre-treated A549 cells. The infectious buffer then was used to infected 293T cells and MDCK to quantify the remained virus particles using luciferase assay and plaque assay, respectively (Figures 3D,E). Interestingly, both luciferase activity and plaque forming units-dependent virus replication showed high level of virus particles in case of the infectious buffer that collected from EMT-104 and EMT-305 treated A549 cells. This result indicates that pretreatment with chemical inhibitors EMT-104 and EMT-305 upset virus entry during the primary infection resulted in high concentration of virus partials in the rest of the infectious buffer.

### Selected chemical compounds reduce interferon production in infected cells

**Figure 4:**
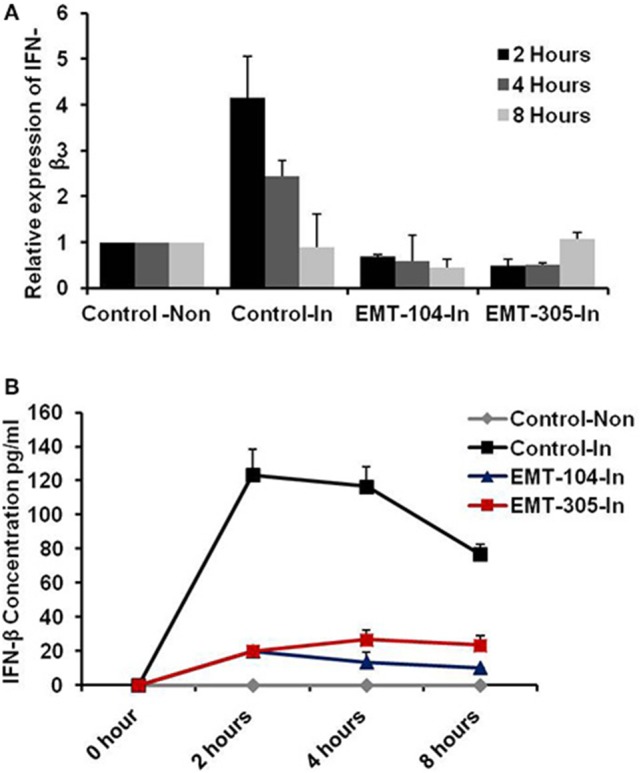
The active compounds disturb the production of IFN-β from infected cells. **(A)** Relative expression of IFN-β on pre-treated and infected A549 cells compared to control infected and noninfected cells. **(B)** Concentration of IFN-β production from infected cells that pre-treated with the active chemical inhibitors indicated by pm/ml. Error bars indicate the standard deviation (*SD*) of three different replicates. The data are represented of three independent experiments.

To further investigate the ability of selected compounds to inhibit virus entry, the expression of interferon beta (IFN-β) and its corresponding protein secretion were detected in pre-treated cells upon 2, 4, and 8 h post-infection. Expectedly, the relative expression of IFN-β was increased in control-infected cells upon 2 h post-infection till the fourth hour (Figure 4A). Interestingly, the production level of IFN-β has been interrupted in control-infected cells upon 8 h post-infection indicating the ability of virus particles to block IFN-β signaling pathway (Figure 4A). Conversely, on pre-treated and infected cells, the relative expression of IFN-β was reduced in all different time point that indicates the lacking of IAV to stimulate the IFN-β pathway and suggests the ability of indicated compounds to disturb virus entry (Figure 4A). Likely, the concentration of IFN-β protein was increased up to 120 pm/ml in 2 h post-infection and then was gradually decreased on control-infected cells (Figure 4B). Whereas the IFN-β concentration was constant in different time point following infection in pre-treated cells (Figure 4B). These findings demonstrate that treatment of A549 cells with the selected chemical inhibitors disturb the virus entry confirmed by lacking of IFN-β production from infected cells.

### The possible interaction between ligand and receptor by molecular docking investigation

**Figure 5:**
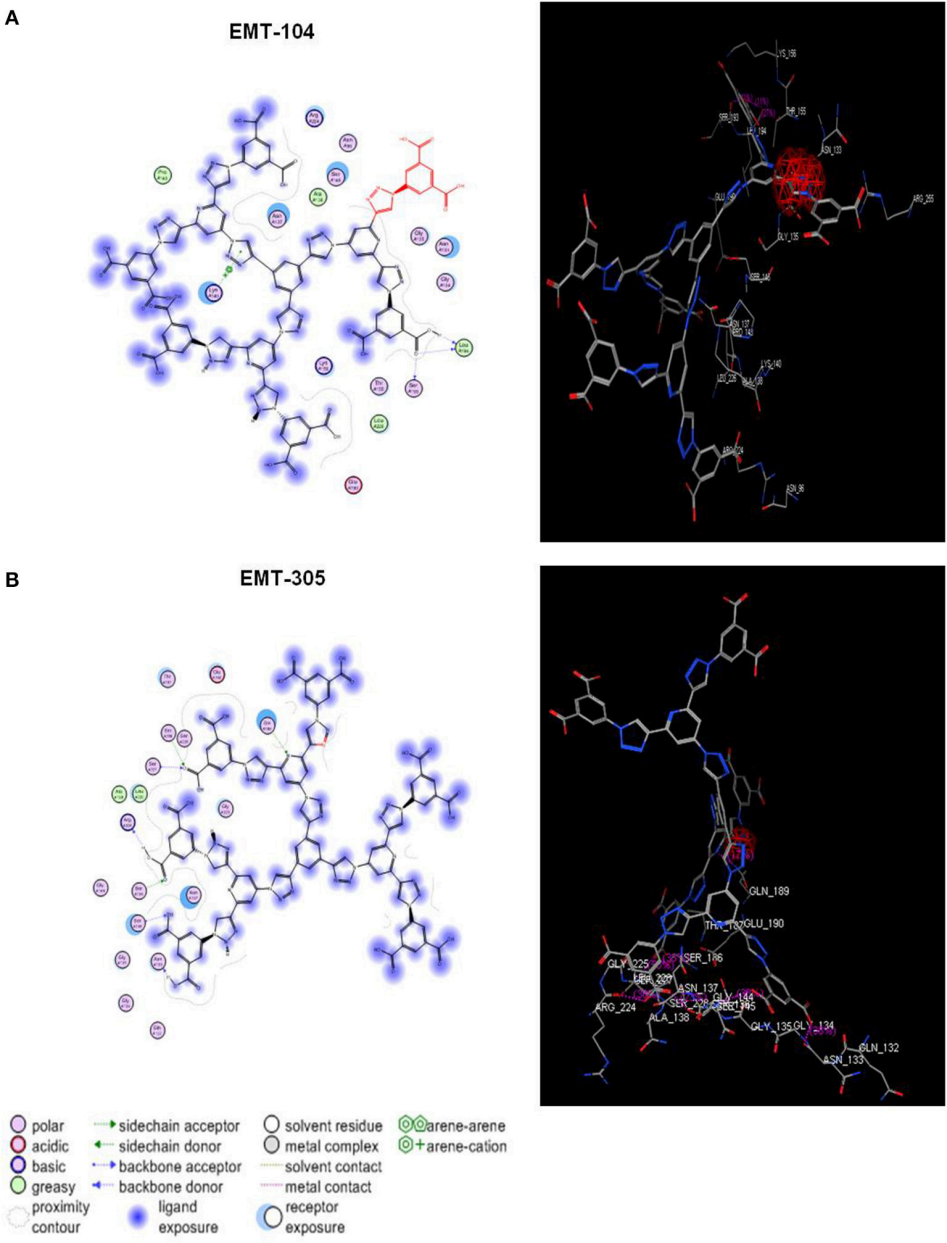
Molecular docking analysis of EMT-305 and EMT-100 compounds with hemagglutinin. **(A)** The molecular docking indicates the binding affinity of synthesized compound EMT-104 and hemagglutinin in host cell receptor and clarifies the seeding region of the possible interaction between the inhibitor EMT-104 and sialic acid. **(B)** The molecular docking of synthesized compound EMT-305 and hemagglutinin in host cell reveals the binding affinity receptor and seeding region of the possible interaction between the inhibitor EMT-305 and sialic acid.

The possible interaction between the inhibitors (EMT-104 and EMT-305) and hemagglutinin protein has been further investigated by molecular docking analysis. The docking analysis showed possible interaction between the inhibitors EMT-104 and EMT-305 with sialic acid in host cell hemagglutinin (Figures 5A,B, respectively). Taken together these results suggest that the chemical inhibitors disturb virus entry may via interaction with sialic acid in host cell receptors.

## Discussion

Our findings here indicate novel and active water soluble compounds (EMT-104 and EMT-305) against IAV infection without detectable toxic effect on cell viability and cell proliferation. These identified compounds have the ability to disturb virus entry via binding with sialic acid in cell receptors and decrease the connection chance of viral hemagglutinin and host cell surface. Recently, solubility of chemical compounds in aqueous buffer has become a critical issue in drug discovery to prevent several barriers in biological challenge assays. For instance, Dimethyl sulfoxide (DMSO) is an organic compound with a median lethal dose higher than ethanol usually used at concentration of 30 mM to dissolve hydrophobic compounds (Papaneophytou et al., 2013). Noteworthy, when absorbed through skin, DMSO causes contamination and unexpected harmful cytotoxic effects in cellular function resulted in suppression of cell proliferation (Oz et al., 2012). Thus, the current data provide novel and active candidates that can inhibit IAV entry to host cells with high solubility in aqueous buffer. Accordingly, virus particles in the rest of infectious media was quantified upon the primary infection to investigate the possible interruption of virus entry to treated cells. Interestingly, both luciferase assay and plaque assay showed high concentration of virus particles remained in infectious media used to infect A549 cells that treated with both EMT-305 and EMT-104 compared to control treated cells. Several studies demonstrate that IAV enters the host through the binding between viral HA and sialic acid as an initial receptor (Wagner et al., 2002). Subsequently, the viral nucleocapsids transfer to the host nucleus for the primary transcription to produce necessary proteins for replication such as PB1 protein. Once the initial proteins are made, eight positive sense cRNA strands are transcribed from the eight negative sense RNA segments which produce again a negative sense RNA and translated to major virus proteins (Nayak et al., 2004). Then the proteins assemble with the other matrix protein (M1), and begin the budding process (Pinto and Lamb, 2006a,b; Bouvier and Palese, 2008). Finally, viral neuraminidase cleaves the binding site between hemagglutinin and cell receptors to facilitate the virus release (Schmitt and Lamb, 2004; Sidorenko and Reichl, 2004). Notably, viral NP protein is a major component of ribonucleoprotein complex which plays the critical role in RNA transcription and viral replication. Excluding the possibility of viral escape mutation, targeting of NP protein disturbs transcription, replication and intracellular trafficking of the virus genome (Portela and Digard, 2002; Turrell et al., 2013). On the other hand, once the virion enters the cells, a significant induction of RIG-I expression is stimulated and a critical signaling cascade is initiated followed by transcription of IFN-β (Wagner et al., 2002; Opitz et al., 2007; Khalil, 2017). Other studies have been shown that viral NS1 protein inhibits the innate and adaptive immune response by multiple mechanisms. One of these mechanisms is the interruption of IFN-β transcription via direct association with RIG-I protein (Hatada et al., 1999; Pichlmair et al., 2006). Therefore, the level of IFN-β production from infected cells is a key biomarker for virus entry and viral successful replication. Likely, the current data showed that IFN-β production was strongly reduced in cells that pre-treated with indicated compounds in a time course experiment. These findings further confirm that the selected chemical compounds have the ability to disturb IAV entry to the host. One explanation of this disturbing of virus entry in treated cells is the interaction between indicated compounds and cellular hemagglutinin receptors through binding with sialic acid. The synthesized compounds EMT-104 and EMT-305 were investigated for the binding affinity of sialic acid receptor for the purpose of lead optimization and to find out the interaction between the indicated compounds and the sialic acid receptor. Sialic acids (Sias) are a family of nine carbon monosaccharides that are usually found on the outermost capping positions of glycans that are linked to cell-surface glycoproteins and glycolipids (Schauer, 2000). Interestingly, our docking analysis that investigate the possible interaction between the chemical inhibitors and hemagglutinin in cell receptors reveals high priority of the binding between these compounds and sialic acid in host cells. Together, these findings reveal that the active selected compounds inhibit IAV infection in treated cells and reduced viral replication via disturbing of virus entry. interestingly, these active compounds are able to be dissolved in water therefore the cytotoxic effect is negligible on treated cells. Further, these active compounds need to be investigated *in vivo* by intratracheal delivering of selected inhibitors into the mice model following by virus inoculations and monitoring the lung virus loads.

## Authors contributions

HK planned and designed the study. TE prepared the chemical inhibitors. The development of experiments and data analysis was assessed by HK, AIE, IE, AAE, and ME. HK wrote the manuscript.

### Conflict of interest statement

The authors declare that the research was conducted in the absence of any commercial or financial relationships that could be construed as a potential conflict of interest.
